# Nogo-A and LINGO-1: Two Important Targets for Remyelination and Regeneration

**DOI:** 10.3390/ijms24054479

**Published:** 2023-02-24

**Authors:** Ilias Kalafatakis, Fevronia Papagianni, Konstantinos Theodorakis, Domna Karagogeos

**Affiliations:** 1 Department of Basic Science, Medical School, University of Crete, 70013 Heraklion, Greece; 2 Institute of Molecular Biology and Biotechnology—FORTH, 70013 Heraklion, Greece; 3 Department of Biology, University of Crete, 70013 Heraklion, Greece

**Keywords:** multiple sclerosis, demyelination, regeneration, remyelination, Nogo-A, LINGO-1

## Abstract

Multiple sclerosis (MS) is an inflammatory disease of the central nervous system (CNS) that causes progressive neurological disability in most patients due to neurodegeneration. Activated immune cells infiltrate the CNS, triggering an inflammatory cascade that leads to demyelination and axonal injury. Non-inflammatory mechanisms are also involved in axonal degeneration, although they are not fully elucidated yet. Current therapies focus on immunosuppression; however, no therapies to promote regeneration, myelin repair, or maintenance are currently available. Two different negative regulators of myelination have been proposed as promising targets to induce remyelination and regeneration, namely the Nogo-A and LINGO-1 proteins. Although Nogo-A was first discovered as a potent neurite outgrowth inhibitor in the CNS, it has emerged as a multifunctional protein. It is involved in numerous developmental processes and is necessary for shaping and later maintaining CNS structure and functionality. However, the growth-restricting properties of Nogo-A have negative effects on CNS injury or disease. LINGO-1 is also an inhibitor of neurite outgrowth, axonal regeneration, oligodendrocyte differentiation, and myelin production. Inhibiting the actions of Nogo-A or LINGO-1 promotes remyelination both in vitro and in vivo, while Nogo-A or LINGO-1 antagonists have been suggested as promising therapeutic approaches for demyelinating diseases. In this review, we focus on these two negative regulators of myelination while also providing an overview of the available data on the effects of Nogo-A and LINGO-1 inhibition on oligodendrocyte differentiation and remyelination.

## 1. Introduction

Myelin, produced by specialized myelinating glial cells (oligodendrocytes (OLs) in the CNS and Schwann cells (SCs) in the PNS, provides mammals with an evolutionary advantage that insulates the axon, provides trophic support, and ensures the rapid and efficient propagation of action potentials along its length. Its disruption, termed demyelination, may occur as a consequence of aging, from genetic alterations in genes encoding myelin proteins (dysmyelination), or from an inflammatory response against myelin producing cells, as is the case in Multiple Sclerosis (MS). The extent of demyelination is consistent with the neurological decline in the previously mentioned conditions, including impairments in motor and cognitive functions [1,2].

One of the most common demyelinating diseases of the CNS is multiple sclerosis (MS), affecting 2.8 million people globally. Previous studies have shown that immunity-associated genes are able to increase the risk of MS, confirming a role of autoimmune mechanisms in the pathogenesis of the disease. In MS, there is infiltration of the activated mononuclear cells in the CNS and microglia activation in the lesion site, which trigger an inflammatory cascade, resulting in demyelination and axonal injury. Non-inflammatory mechanisms, such as the generation of oxygen and nitrogen reactive species, mitochondrial damage, and intra-axonal accumulation of calcium, also play an important role in axonal degeneration. These mechanisms lead to impaired energy production and progressive proteolytic degradation of cytoskeleton proteins resulting in further axonal degeneration and neuronal loss [1]. Remyelination is the generation of new myelin sheaths that acts as a homeostatic repair process and involves the recruitment of oligodendrocyte precursor cells (OPCs) at the lesion site. These OPCs will then differentiate to mature myelinating OLs. Nevertheless, in most cases, remyelination is insufficient as the damaged fibers are not able to function properly. The reason is that either few OPCs are recruited in the affected area due to decreased proliferation or migration, or alternatively, the recruited OPCs may not properly differentiate to fully myelinating OLs. Indeed, it has been shown that OPC maturation can stall after the premyelinating stage; therefore, these differentiating oligodendrocytes can contact or even enwrap axons but cannot produce compact myelin. As a result, axonal degeneration ensues, as observed in advanced stages of MS [2,3,4,5].

Current MS therapies include anti-inflammatory and immunomodulatory agents, drugs that inhibit inflammation, aiming to reduce the frequency of relapses and tissue injury associated with acute inflammation. Even though these drugs have the ability to slow the evolution of the disease [6,7,8,9,10], they do not appear to stop tissue loss or promote remyelination and axonal repair [11], while their effects on long-term disability progression are still controversial. Compounds that may promote OPC proliferation/migration or differentiation to fully myelinating OLs may provide the means of more efficient repair [5]. One of the main reasons for insufficient remyelination is the gradual disappearance of growth promoting factors and the enhanced appearance of growth inhibitors, such as Nogo-A and LINGO-1, which are our focus in this review [12].

## 2. Nogo-A Expression, Structure and Function

Nogo protein is encoded by the RTN4 gene [13], which is part of the reticulons RTN4 ER family of genes [14] and has three isoforms [15]. Nogo-A (200 kDa) is expressed in the CNS. In adult mice, Nogo-A is mainly expressed in oligodendrocytes [16,17] but also in the neurons [18] and microglia [19] in brain areas such as the hippocampus, spinal cord, and cerebellum [18]. Nogo-B (55kDa) is expressed in cardiac myocytes [18], vascular and endothelial cells [20,21], and Nogo-C (25 kDa) is expressed in many cells such as neurons, liver cells, and muscle cells [22,23]. All three isoforms have the same conserved C-terminal (RHD) but different N-terminal domains [24].

In this review, we will focus on Nogo-A since it is one of the most important myelin-associated inhibitors of axonal regeneration [13,25,26,27,28] and neuronal plasticity in the CNS [16,17,18]. As mentioned above, during development, Nogo-A is expressed mainly by neurons, where it plays an important role in the migration of cells in neural tubes [29], the interaction between axons and oligodendrocytes [16], axon guidance by the repulsion of growing fibers [30], and angiogenesis [31]. Additionally, it is highly expressed in growing neurites [32], modulating their fasciculation and branching [33]. In the adult CNS, it is mainly expressed by oligodendrocytes [17] in the innermost and outer myelin membrane [18], and it is an inhibitor of axonal growth and plasticity [34], stabilizing the CNS wiring [26]. It is a long transmembrane protein consisting of 1192 amino acids localized in the plasma and the endoplasmic reticulum (ER) membrane [35,36].

**Figure 1:**
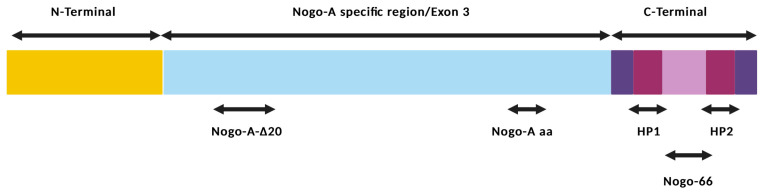
Structural representation of Nogo-A showing its 3 functional domains important for neurite growth inhibition: Nogo-66 domain, which is located near C-terminal between two hydrophobic domains (HP1, HP2); Nogo-A-Δ20, which is located near N-terminal of the protein; and the recently identified Nogo-A aa domain. Created with BioRender.com, accessed on 10 February 2023.

There are two main functional domains of Nogo-A [37] that are important for neurite growth inhibition: Nogo-A-Δ20 (544–725 amino acid residues), which is included in the Nogo-A specific region/Exon 3 and is located near the N-terminal of the protein, and Nogo-66 (1055-1120 amino acid residues), which is located near the C-terminal between two hydrophobic domains (HP1, HP2) [13]. Nogo-A-Δ20 is important for the inhibition of axonal spreading and regrowth while modulating neuron migration in development. Nogo-66, which is anchored on the membrane surface of oligodendrocytes, is responsible for growth cone collapse (Figure 1) [38]. Recently, a new functional domain of Nogo-A was identified, Nogo-A aa (846–861 amino acids residues). This region is responsible for the inhibition of axonal growth and for the promotion of inflammatory pain [39].

## 3. Nogo-A Interactions and Signaling

**Figure 2:**
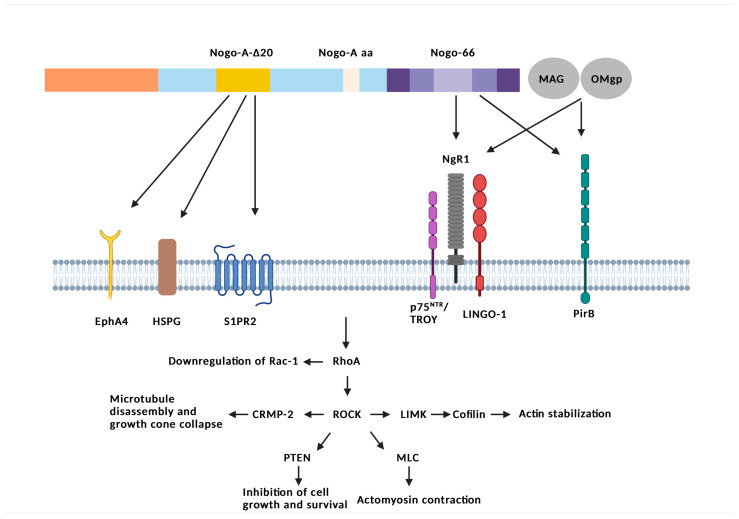
Schematic representation of Nogo-A receptors and signaling. The key to the inhibitory action of Nogo-A is the binding of Nogo-66 and Nogo-A-Δ20 domains with different receptors. NgR1 is the main receptor of Nogo-66. Important for the signaling of the receptor NgR1 is its connection to p75 or/and its connection to TROY and LINGO-1. Except for Nogo-A, MAG and OMgp can also bind to NgR1, having similar effects regarding the inhibition of axonal regeneration and sprouting. Another receptor that interacts with these three myelin-associated proteins is PirB. Except for Nogo-66, there are interactions between the Nogo-A-Δ20 region and other receptors such as S1PR2, HSPGs, and EphA4. The main pathway, activated by the interactions of Nogo-66 with NgR1 and Nogo-A-Δ20 with S1PR2, HSPGs, and EphA4, is the Rho GTPase pathway and, simultaneously, the downregulation of activated Rac1. The RhoA gene activates ROCK, which promotes actomyosin contraction through the increase of MLC phosphorylation. RhoA-ROCK signaling also promotes the stabilization of actin filaments through the induction of LIMK and inactivation of cofilin. ROCK interacts with CRMP-2. ROCK-mediated phosphorylation of CRMP-2 blocks its ability to bind to tubulin, thereby leading to inhibition of microtubule assembly and growth cone collapse. Lastly, ROCK phosphorylates PTEN, inhibiting cell growth and survival. Created with BioRender.com, accessed on 10 February 2023.

The key to the inhibitory action of Nogo-A is the binding of Nogo-66 and Nogo-A-Δ20 domains with different receptors, such as the NgR1 (The Nogo Receptor) or the S1PR2 (sphingosine-1-phosphate receptor 2) [16,40], as depicted in Figure 2. NgR1 is the main receptor of Nogo-66, also encoded by the RTN4 gene, and is expressed in the neurons and axons of the CNS, microglia, and astrocytes [41]. NgR1 is a glycophosphatidylinositol-anchored protein (GPI), so it does not have an intracellular component [41], which is why it needs a transmembrane coreceptor like LINGO-1 to convert the signal from the extracellular to the cytosolic environment [42]. For the signaling of the receptor NgR1, its connection to p75, a neurotrophic receptor and/or its connection to TROY, a tumor necrosis factor [43,44], is important. Except for Nogo-A, MAG (myelin-associated glycoprotein) and OMgp (oligodendrocyte myelin glycoprotein) can also bind to NgR1, having similar effects regarding the inhibition of axonal regeneration and sprouting (Figure 2) [45,46,47].

Another receptor, which interacts with these three myelin-associated proteins, is the paired immunoglobulin-like receptor B (PirB, Figure 2) [48]. PirB plays an important role in plasticity in the visual cortex [49]. Recently, another receptor of Nogo-A has been found using CRISPR screening, the brain angiogenesis inhibitor 1, BAI1 (ADGRB1) [50]. Except for Nogo-66, there are interactions between the Nogo-A-Δ20 region and other receptors such as sphingosine-1-phosphate receptor 2 (S1PR2), heparan sulfate proteoglycans (HSPGs), and ephrin type-A receptor 4 (EphA4), which are important to NSC apoptosis via JNK MAPK pathway activation [51]. However, the main pathway, activated by the interactions of Nogo-66 with NgR1 and Nogo-A-Δ20 with S1PR2, HSPGs, and EphA4, is the Rho GTPase pathway [52] and, simultaneously, the downregulation of activated Rac1 [53]. The Ras homolog gene family member A, the RhoA gene, activates the Rho-associated protein kinase, ROCK, which promotes actomyosin contraction through the increase of myosin light chain (MLC) phosphorylation [54]. RhoA-ROCK signaling also promotes the stabilization of actin filaments through the induction of the LIM kinase-dependent phosphorylation (LIMK) and inactivation of cofilin [55,56]. ROCK also interacts with collapsin response mediator protein-2 (CRMP-2). CRMP-2 is a microtubule-binding protein that induces axon growth through the promotion of microtubule assembly. ROCK-mediated phosphorylation of CRMP-2 blocks its ability to bind to tubulin, thereby leading to the inhibition of microtubule assembly and growth cone collapse [57]. Last but not least, ROCK phosphorylates the dual protein/lipid phosphatase PTEN, which is a tumor suppressor able to inhibit cell growth and survival [58]. Thus, the signaling cascade includes reduced growth of actin filaments [59], the collapse of the growth cone [60], destabilization of microtubules, and downregulation of growth genes in the neuronal cell body, leading to axon growth inhibition and destabilization of synapses [41,61]. It is important to mention that CRMP-2 is inhibited during the progression of experimental autoimmune encephalomyelitis (EAE) in degenerating axons [62]. Moreover, expression of NgR1 is increased in astrocytes and microglia in actively demyelinating lesions of MS. Thus, this may indicate an alternative modulation of inflammation via the Nogo-A/NgR signaling pathway (Figure 2) [63]. Last but not least, as mentioned in the previous section, a new functional domain of Nogo-A was recently identified (Nogo-A aa). This domain was characterized as a novel extra ligand of NgR1 that is able to activate the downstream signaling pathways inhibiting axonal growth and promoting inflammatory pain [39].

## 4. Nogo-A Is Implicated in Many Neurodegenerative Diseases

Nogo-A plays a critical role in many neurodegenerative disorders, such as amyotrophic lateral sclerosis (ALS), temporal lobe epilepsy (TLE), and Alzheimer’s disease (AD). Previous studies have shown that Nogo-A expression is increased in both ALS and TLE patients [64,65]. Nogo-A also plays an important role in AD pathogenesis. Particularly, Nogo-A receptors modulate the generation of amyloid β-protein (Aβ), which is thought to be a major cause of AD [66]. Nogo-A is also crucial in other diseases like glioblastoma and schizophrenia. The Nogo-A/NgR pathway plays an important role in regulating cancer stem-like cells (CSCs) derived from glioblastoma affecting cell viability, cell cycle entry, invasion, and tumor formation [67]. Regarding the role of Nogo-A in schizophrenia, it is proposed that abnormal Nogo-A expression or NgR mutations may be characterized as genetic risks for neuropsychiatric disorders of presumed neurodevelopmental origin, such as in the case of schizophrenia. Previous studies in a mouse model of genetic Nogo-A deficiency have shown that Nogo-A deletion may lead to schizophrenia-like abnormalities [68]. Moreover, blocking Nogo-A leads to retinal and visual recovery [69]. For decades, studies have shown that blocking Nogo-A has a therapeutic role in MS [70,71], Parkinson’s disease (PD) [72], spinal cord injury (SCI) [73,74], and stroke [75]. Recently, it was demonstrated that deletion of the Nogo-A gene is able to modulate inflammatory diseases through the regulation of cytokines [76]. In this review, we will focus on the role of Nogo-A inhibition in demyelinating disorders such as MS.

## 5. The Role of Nogo-A Inhibition in Demyelination/Remyelination

One promising approach for the promotion of remyelination is to design antibodies against the Nogo-A protein. Administration of anti-Nogo-A in vivo not only neutralizes the inhibitory role of Nogo-A but also enhances neurite outgrowth and neuronal survival. Additionally, no differences between the two genders and no side effects, in general, were observed [77,78].

Many studies in MS animal models support the idea that inhibition of Nogo-A, either by injections with specific antibodies or by depletion of the Nogo-A gene, could be a therapeutic approach for MS. To mimic demyelinating disorders like MS, there are three main animal models used: Cuprizone-induced, LPC (lysolecithin)-induced, and experimental autoimmune encephalomyelitis (EAE) [79], the last being the most widely used for this purpose [80]. In studies on the EAE model, the suppression of Nogo-A through injections with anti-Nogo-A antibodies showed promising results [71,81]. More specifically, they showed lower clinical scores of EAE, suggesting a decreased severity of the disease [81], while reduced levels of inflammation, demyelination, and axonal degeneration and a generally slower disease progression were also observed [71]. Additionally, in vivo and in vitro experiments in Nogo-A deficient mice through RNA silencing have shown enhanced axonal repair [81]. These results confirm the idea that axonal remyelination, as mentioned before, is the key to improving the clinical state of MS [70,82]. Moreover, depletion of the Nogo-A gene in LPC models of demyelination and stroke leads to enhanced axonal plasticity and fiber growth [83]. A study focusing on the expression pattern of Nogo-A during EAE progression showed a reduction of Nogo-A mRNA expression at preclinical and acute phases, followed by an increase during the chronic phase. The expression of Nogo-A protein was also found to increase in the chronic phase. This increase is correlated with an increase of cortical NgR protein and mRNA levels during the same time point, suggesting that there is active regulation of both Nogo-A and its receptor in EAE lesions [84]. Furthermore, the comparison of the expression of Nogo-A mRNA and, consequently, Nogo-A protein with the levels of growth-associated protein GAP43 (neuromodulin) in neurons has shown an interesting connection [85]. More specifically, in the primary phase of EAE, Nogo-A is downregulated while neuromodulin is upregulated. However, in the chronic demyelination stage, the opposite happens. Moreover, suppression of Nogo-A was associated with enhanced plasticity and the regrowth of fibers and improvement in locomotion via behavioral approaches in models of spinal cord or brain injury [26,27]. Finally, similar experiments on monkeys showed that neutralization of Nogo-A promotes axonal sprouting and functional recovery after spinal cord injury (Table 1) [77,78].

In addition to these preclinical studies, it is known that in MS patients with chronic demyelinated lesions, Nogo-A is upregulated. Some antibodies for Nogo-A were found in the blood serum of patients, just as they are found in healthy people [86]. The preclinical studies using different models of demyelinating diseases and data from MS patients suggest Nogo-A antibodies as a potential therapeutic approach for the treatment of relapsing-remitting MS (RRMS) and/or progressive forms of MS. Two Phase I studies for antibodies against Nogo-A were conducted in patients with RRMS (ClinicalTrials.gov, NCT01424423 and NCT01435993). Both studies, involving a limited number of patients, were terminated, and their results were not fully published [87].

Additionally, two other Phase I studies focusing on two other CNS diseases were successfully completed, testing the acute safety, tolerability, and pharmacokinetics of anti-Nogo-A antibodies. The first study focused on the intrathecal administration of anti-Nogo-A antibodies for 30 days in patients with SCI (NCT00406016). This study was the first one investigating anti-Nogo-A delivery for the treatment of acute SCI. It was shown that anti-Nogo-A (ATI335) was well-tolerated, and its delivery, particularly via bolus injection, is a viable method of drug administration in acute SCI. The small number of patients and the fact that this was an open-label study resulted in a limited ability to draw safe conclusions regarding drug efficacy in improving neurological recovery after SCI. The second study focused on the administration of doses of intravenously infused antibodies in patients with ALS(NCT00875446). In this study, it was shown that a humanized monoclonal antibody against Nogo-A (ozanezumab) was well tolerated. The differences observed regarding functional endpoints and the colocalization of ozanezumab in skeletal muscle were promising. These observations, together with the lack of emerging safety signals, support the planning of future clinical trials aiming to produce more successful outcomes in the treatment of neurodegenerative diseases like MS and ALS. Both clinical studies showed excellent safety and tolerability of the Nogo-A antibody treatment [88,89,90].

However, additional studies should be performed with a higher number of patients suffering from demyelinating diseases for more accurate and safe results regarding the role of Nogo-A antibodies as a promising therapeutic agent. Last but not least, it is worth mentioning that until today, one of the most important concerns regarding anti-Nogo-A administration is its potentially limited access to the CNS through the BBB [91].

## 6. LINGO-1 Expression, Structure and Function

**Figure 3:**
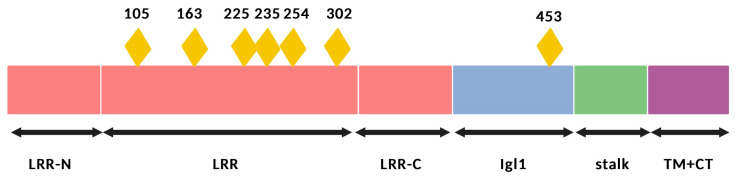
Structural representation of LINGO-1. This protein consists of an extracellular domain. The yellow rhombi represent the occupied N-linked glycosylation sites. The domain also contains 12 leucine-rich repeat (LRR) motifs with N- and C-terminal caps and an immunoglobulin (Igl1) domain necessary for its biological activity. Created with BioRender.com, accessed on 11 February 2023.

Neurite outgrowth inhibitor receptor-interacting protein (LINGO-1), containing leucine-rich repeats and Ig domains, has served as a potent negative regulator of oligodendrocyte differentiation and myelination [92]. LINGO-1, which is encoded by the LRRN6A gene, is a type I transmembrane composed of 614 amino acids. LINGO-1 consists of an extracellular domain, which is heavily glycosylated with 12 leucine-rich repeat (LRR) motifs encompassing N- and C-terminal caps and an immunoglobulin (Igl1) domain [93,94]. The Ig domain of LINGO-1 is necessary and sufficient in mediating its biological function [95,96]. The protein forms a ring-shaped tetramer in which the Ig domain makes contact with the N-terminal LRR sequences from an adjacent LINGO-1 molecule (Figure 3) [97].

The LINGO-1 structure is highly conserved. A rostral to the caudal gradient of LINGO-1 expression in the adult CNS showed the highest levels in the cerebral cortex, the hippocampus, the amygdala, and the thalamus, with a more basal level of expression across the remainder of the brain, while the lowest levels of LINGO-1 expression are observed in the spinal cord [98]. Previous studies have shown that LINGO-1 mRNA is expressed in the CNS throughout embryonic and postnatal stages [42,43,92,93,99,100,101,102]. More specifically, LINGO-1 is expressed on oligodendrocytes and neurons [92,102], while it is associated with the Nogo-66 receptor (NgR1) complex [41,42,43,44,46,103,104].

## 7. LINGO-1 Interactions and Signaling

**Figure 4:**
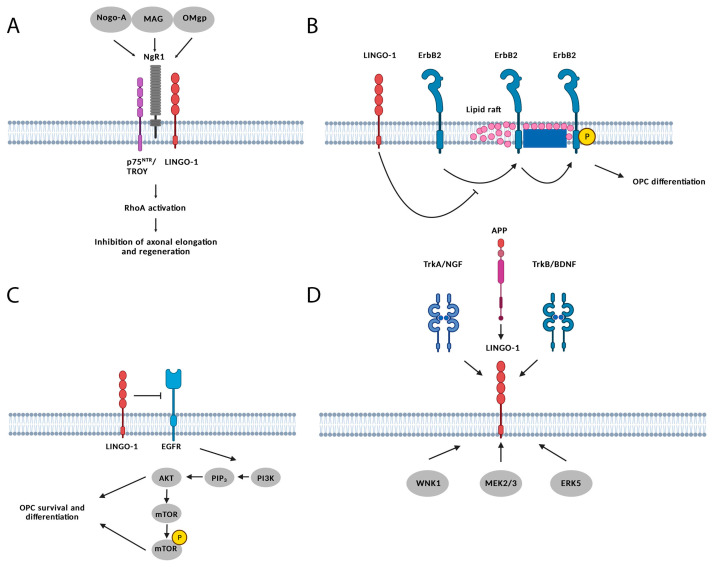
Schematic representation of LINGO-1 interactions and signaling. (**A**) LINGO-1 forms a complex with NgR1 and P75/TROY receptors. This complex is enabled when bound to Nogo-A, OMgp, and MAG and leads to the activation of the RhoA pathway, resulting in the inhibition of axonal regeneration and sprouting. (**B**) LINGO-1 binds to ErbB2, and as a result, ErbB2 does not translocate to the lipid rafts on oligodendroglial membranes. Phosphorylated ErbB2 in lipid rafts induces OPC differentiation. Therefore, LINGO-1 prevents ErbB2 from inducing OPC differentiation. (**C**) LINGO-1 directly inhibits EGFR signaling, thus preventing the activation of intracellular PI3K. Without PI3k activation, PIP2 cannot be further phosphorylated to its active form, and as such, AKT and m-TOR remain in their inactive forms. Both activated AKT and m-TOR have been shown to promote OPC survival and differentiation. As such, LINGO-1 inhibits OPC differentiation. (**D**) LINGO-1 also interacts with NGF and its receptor TrkA, BNDF and its receptor TrkB, and APP. It also interacts with WNK1, MEK 2/3, ERK5, and other proteins implicated in neurological disorders. Created with BioRender.com, accessed on 10 February 2023.

As mentioned above, NgR1 binds to the following myelin-associated inhibitors: Nogo-A [13,37], MAG [45,105], and OMgp [46]. The binding of a myelin-associated inhibitor to the NgR1 complex induces an intracellular signaling cascade through LINGO-1 and p75NTR/TROY [106]. Protein kinase C and Ca^2+^-dependent or -independent activation of a small RhoA guanosine triphosphatase (RhoA GTPase) molecule is the key intracellular event leading to the inhibition of axonal regeneration, elongation, and oligodendrocyte differentiation [107,108] (Figure 4A) [109].

Other than its role as a negative regulator of myelination, LINGO-1 is also involved in the regulation of neural apoptosis by inhibiting WNK3 kinase activity. LINGO-1 is able to interact with different co-factors and coreceptors, leading to the activation of signaling pathways involved in the regulation of neuronal survival, axon regeneration, oligodendrocyte differentiation, and myelination processes in the brain. LINGO-1 binds to epidermal growth factor receptor 2 (ErbB2) and inhibits its translocation to the lipid rafts on oligodendroglial membranes. In the absence of LINGO-1, ErbB2 is modulated in lipid rafts by local kinases and phosphatases and, in this way, induces OPC differentiation (Figure 4B) [110,111,112]. Moreover, LINGO-1 is able to inhibit epidermal growth factor receptor (EGFR) signaling, preventing the activation of intracellular phosphoinositide 3-kinase (PI3K) [113]. Without PI3k activation, PIP2 cannot be further phosphorylated to its active form, and, as such, AKT and m-TOR remain inactive. Since both activated AKT and m-TOR have been shown to promote OPC survival and differentiation, LINGO-1 prevents OPC differentiation (Figure 4C) [114].

LINGO-1 also interacts with the nerve growth factor (NGF) and its receptor tropomyosin receptor kinase A (TrkA), brain-derived neurotrophic factor (BDNF) and its receptor tropomyosin receptor kinase B (TrkB), and amyloid precursor protein (APP). It also interacts with proteins that are implicated in neurological and psychiatric disorders, such as WNK lysine-deficient protein kinase 1 (WNK1), mitogen-activated protein kinase 2/3 (MEK 2/3), extracellular signal-reduced kinase 5 (ERK5), and others(Figure 4D) [98].

## 8. LINGO-1 Is Implicated in Many Neurodegenerative Diseases

The expression of LINGO-1 is increased in many CNS diseases. This increase is associated with CNS injury and neuronal cell death, suggesting that LINGO-1 may play an important role during cell injury response. LINGO-1 is implicated in glaucoma, PD, SCI, traumatic brain injury, MS, essential tremor, as well as AD and epilepsy due to its role in the inhibition of axonal outgrowth, neuronal death, oligodendrocyte differentiation, and myelination [98]. More specifically, increased LINGO-1 expression is associated with glaucoma, which is characterized by the degeneration of retinal ganglion cells (RGCs) and their axons. LINGO-1 inhibition leads to the neuroprotection of damaged RGCs in well-established models of chronic glaucoma and acute optic nerve transection, possibly through inhibition of RhoA activation or activation of the Akt survival signaling pathway [115]. Increased LINGO-1 expression is also observed in the substantia nigra of PD patients and animal models of PD. LINGO-1 is expressed in midbrain dopaminergic (DA) neurons in both human and rodent brains. Depletion of LINGO-1 in mice is associated with increased survival of DA neurons and reduced behavioral abnormalities in PD models due to the activation of the EGFR/Akt signaling pathway [113]. LINGO-1 is also implicated in SCI since it is detected in the axonal tracts of rat spinal cords following injury, with an increase of LINGO-1 mRNA levels observed 14 days post-injury [42]. LINGO-1 expression is also associated with traumatic brain injury, which involves cell death in the cerebral cortex and hippocampus [116]. These areas highly express LINGO-1 during both development and adulthood [42,93], while RhoA pathway activation is responsible for the lack of regeneration of damaged axons [117]. Regarding the role of LINGO-1 in the pathophysiology of AD, LINGO-1 is able to bind to amyloid precursor protein and regulate its processing by increasing the production of amyloid-β peptide [100]. LINGO-1 is able to regulate the amount of amyloid precursor protein available for processing [118]. LINGO-1 also plays an important role in neurological disorders like tuberous sclerosis, focal cortical dysplasia, and TLE, which all include seizures as a common symptom. Previous studies showed that both protein and mRNA levels of Nogo-A, NgR, LINGO-1, TROY, and RhoA increased in the cortex of tuberous sclerosis and cortical dysplasia patients. LINGO-1 and TROY were also expressed in the reactive astrocytes of these patients, suggesting an important role of Nogo-A and its signaling complex LINGO-1/NgR/TROY in the development and progression of seizure activity [119]. Last but not least, LINGO-1 and its signaling partners are also implicated in the pathophysiology of schizophrenia and a number of other neuropsychiatric disorders like depression, attention-deficit hyperactivity disorder, autism spectrum disorder, anxiety, post-traumatic stress, and drug addiction mainly due to LINGO-1 activating or blocking pathways leading to negative regulation of myelination and neurite outgrowth [98].

## 9. The Role of LINGO-1 Inhibition in Demyelination/Remyelination

Focusing on the role of LINGO-1 in myelination and demyelination, it is proposed that LINGO-1 has an important role in repressing oligodendrocytes differentiation, as mentioned above [92]. Additionally, extracellular blockage of LINGO-1 function overcomes the myelin inhibitory activity in the spinal cord that prevents axonal regeneration after lesions in rats [120]. Moreover, LINGO-1 inhibition improves dopaminergic neuron activity in a model of Parkinson’s disease and promotes spinal cord remyelination in an experimental model of autoimmune encephalomyelitis [113,121].

LINGO-1 function has also been investigated in animal models of demyelination. The measurement of transcranial magnetic motor-evoked potentials (tcMMEPs) provides an accurate readout of the electrophysiological function of descending tract demyelination/remyelination of the spinal [122]. In rats and mice, tcMMEPs are only transmitted through the ventrolateral funiculus (VLF) region [123,124]. Demyelination increases the latency while decreasing the amplitude of tcMMEPs. In LPC-induced demyelinating lesions of the rat VLF, inhibition of LINGO-1 improves the recovery of tcMMEP amplitude in parallel with increases in the thickness of myelin sheaths [125].

Inhibition of LINGO-1 by using specific antibodies improved remyelination and neurobehavioral deficits in cuprizone-induced demyelination. In this study, levels of MBP, BDNF, and NF200 were increased after the treatment of anti-LINGO-1 in this model, while behavioral studies showed an improvement regarding motor impairments after the same treatment [126]. Another study confirmed that LINGO-1 antagonism is able to promote repair in the EAE model of demyelination through the ability of LINGO-1 antagonists to promote the differentiation of OPCs. The EAE mouse model is characterized by learning and memory deficits occurring in late EAE and decreased expression of MBP in the parahippocampal cortex (PHC) and fimbria-fornix. Administration of the LINGO-1 antibody significantly improved learning and memory in EAE and partially restored MBP in PHC [127].

Anti–LINGO-1 antibody treatments significantly increased the in vivo rate of remyelination in two different models of demyelination (LPC and cuprizone) and enhanced axonal conduction in LPC lesions [125]. Additional studies confirmed the promising results above. More specifically, mice treated with LINGO-1-directed siRNA–chitosan nanoparticles performed better remyelination after ethidium bromide-induced demyelination. Mice treated with LINGO-1 siRNA nanoparticles exhibit enhanced motor performance compared to the non-treated group. LINGO-1 downregulation was associated with signs of repair in histopathological sections as indicated by increased expression of MBP mRNA and protein in the pons and lower levels of caspase-3 activity [128]. Last but not least, studies have shown that LINGO-1 inhibition via RNA interference led to the functional recovery of the EAE mouse model, suggesting higher levels of myelination through Luxol Fast Blue (LFB) staining and better locomotor activity through specific behavioral approaches (Table 2) [129].

All these data propose that a major function of LINGO-1 is to inhibit the differentiation of OPCs, thereby preventing remyelination and that antagonizing the LINGO-1 function represents potential therapeutics for repair in CNS demyelinating diseases. For this reason, there have been studies focusing on designing specific antibodies against LINGO-1 with a potential beneficial role in remyelination and neuroprotection. To induce remyelination, the human monoclonal IgG antibody, opicinumab (BIIB003), was designed in order to inhibit LINGO-1-mediated pathways [130]. Two Phase I clinical studies, including opicinumab administration in healthy volunteers and relapsing MS patients, indicated a tolerable safety profile [131].

A double-blind Phase II study (RENEW) was conducted in patients with acute optic neuritis (AON). After treatment with a standard high dose of methylprednisolone (IVMPS), participants received either 100 mg/kg opicinumab or a placebo within 28 days after symptom onset. Administration of the treatment took place every 4 weeks up to week 20. After week 20, there was a 12-week observation period. The visually evoked potentials (VEPs) assessed the recovery of the affected optic nerve conduction after 24 weeks. Although treatment with opicinumab resulted in amelioration of P100 latency (the time from the stimulus onset to the main positive peak), this difference was not statistically significant [132].

Furthermore, in a new substudy, multifocal VEP measurements were used to examine optic nerve repair. No statistically significant differences towards reduced latency prolongation and increased recovery of VEP amplitude were observed in the active treatment group [133].

Moreover, a two-year follow-up study of the RENEW trial was conducted (RENEWED). Participants of the RENEW clinical study, who received at least one dose of opicinumab or placebo, could join this follow-up trial. Investigation of VEP latency showed that the observed positive trend in the opicinumab group was maintained over two years. However, as mentioned above, this trend was not statistically significant [134,135,136].

Furthermore, a double-blind Phase II SYNERGY trial was conducted, including RRMS and secondary progressive MS (SPMS) patients with relapses who were randomly assigned to either 3, 10, 30 or 100 mg/kg of opicinumab or a placebo treatment. Patients received treatment or a placebo every 4 weeks for 72 weeks in addition to interferon-β1α. In this study, there was no statistically significant beneficial effect regarding treatment with 3, 10, and 100 mg/kg of opicinumab. The only statistically significant beneficial effect was observed in patients who received a dose of 30 mg/kg opicinumab. Although these patients showed an improvement regarding their disability, there was no significant dose linear improvement [137].

Last but not least, a placebo-controlled, randomised, double-blind Phase II trial (AFFINITY) was also conducted, including RRMS and SPMS patients. This study aimed to evaluate the administration of 750 mg of opicinumab, equivalent to a dose of 10 mg/kg. The primary study endpoints (pSE) included the integrated response score already used in the SYNERGY study. After some time, it was announced that the AFFINITY trial failed to meet the pSE [135].

Despite the less-than-promising results, SYNERGY has provided useful information regarding trial design in patients with established MS-related disabilities. This study confirmed a good tolerability and feasibility of treatment with monoclonal antibodies for CNS neurodegenerative diseases. Due to many contradictory results in the literature, future studies must aim to clarify some important aspects regarding LINGO-1. More specifically, it should be clarified whether LINGO-1 is localised to the extracellular cell surface and if it is present in human MS tissue. Additionally, new potential partners involved in downstream LINGO-1 signaling should be identified [135].

The clinical trials conducted so far did not yield promising results as expected based on the preclinical studies, but there are some concerns regarding these trials. They relate to trial design (numbers or characteristics of patients recruited and specific clinical outcomes or time frames), particularly poor penetration of antibodies across the blood–brain barrier due to physical size, and active efflux of antibodies from the CNS compartment. Moreover, additional studies will investigate whether some subpopulations identified might benefit from opicinumab treatment at an optimum dose.

## 10. Concluding Remarks

Current therapeutic approaches for the treatment of MS focus on drugs with anti-inflammatory or immunomodulatory properties. These drugs are useful since they slow disease progression by reducing brain inflammation. However, none of the available therapeutic approaches have been shown to promote tissue recovery. Once CNS damage has occurred, the progression of disability is typically continuous and irreversible. In this direction, new therapeutic approaches should emerge that will promote remyelination and regeneration leading to neurological recovery. As mentioned above, in most MS cases, remyelination is insufficient, failing to restore axonal function, while the presence of undifferentiated OPCs within lesions suggests that OPC differentiation is blocked. For this reason, antagonists of inhibitors of oligodendrocyte differentiation may promote remyelination. In that regard, Nogo-A and LINGO-1 are attractive therapeutic targets. Blocking of Nogo-A or LINGO-1 in different models of demyelination showed some promising results regarding remyelination and recovery. This raises the exciting prospect that antagonists of Nogo-A and LINGO-1 may promote neuronal remyelination, survival, and function, leading to the critical neurological recovery of MS patients in the future. The clinical studies performed regarding the impact of anti-Nogo-A and anti-LINGO-1 in MS-related patients did not yield the promise that was expected, but additional studies and information regarding Nogo-A and LINGO-1 will support the interpretation and planning of future clinical trials aiming for more successful outcomes in the treatment of neurodegenerative diseases like MS.

## Figures and Tables

**Table 1:** Effects of Nogo-A inhibition in animal models.

Models	Method of Nogo-A Inhibition	Effects
EAE	Anti-Nogo-A antibodies	Lower clinical score of EAE Reduced inflammation Reduced demyelination Reduced axonal degeneration
EAE	siRNA against Nogo-A	Enhanced axonal repair
LPC	KO Nogo-A mice	Enhanced axonal plasticity and fiber growth
SCI	Many suppression methods	Enhanced plasticity Fiber regrowth Improvement in locomotion Axonal sprouting Functional recovery

**Table 2:** Effects of LINGO-1 inhibition in animal models.

Models	Method of Lingo-1 Inhibition	Effects
CPZ	Anti-LINGO-1 antibodies	Improved remyelination Increased levels of MBP, BDNF and NF200 Improve motor impairments
EAE	LINGO-1 antagonist	Improved learning and memory Partially restored MBP in PHC
EAE	RNAi	Higher levels of myelination Better locomotor activity
LPC	Anti-LINGO-1 antibodies	Enhanced axonal conduction Increased thickness of myelin sheaths
Ethidium Bromide	LINGO-1–directed siRNA–chitosan nanoparticles	Improved remyelination Better motor performance Higher expression of MBP mRNA and protein and Lower levels of caspase-3 activity
